# Exploring the ATG9A interactome uncovers interaction with VPS13A

**DOI:** 10.1242/jcs.261081

**Published:** 2024-02-22

**Authors:** Alexander R. van Vliet, Harold B. J. Jefferies, Peter A. Faull, Jessica Chadwick, Fairouz Ibrahim, Mark J. Skehel, Sharon A. Tooze

**Affiliations:** ^1^ Molecular Cell Biology of Autophagy, The Francis Crick Institute, London NW1 1AT, UK; ^2^ Proteomics Science Technology Platform, The Francis Crick Institute, London NW1 1AT, UK; ^3^ MRC Laboratory of Molecular Biology, Cambridge CB2 0QH, UK

**Keywords:** ATG9A interactome, Autophagy, VPS13, Lipid trafficking, Mass spectrometry

## Abstract

ATG9A, a transmembrane protein of the core autophagy pathway, cycles between the Golgi, endosomes and a vesicular compartment. ATG9A was recently shown to act as a lipid scramblase, and this function is thought to require its interaction with another core autophagy protein, ATG2A, which acts as a lipid transfer protein. Together, ATG9A and ATG2A are proposed to function to expand the growing autophagosome. However, ATG9A is implicated in other pathways including membrane repair and lipid droplet homeostasis. To elucidate other ATG9A interactors within the autophagy pathway, or interactors beyond autophagy, we performed an interactome analysis through mass spectrometry. This analysis revealed a host of proteins involved in lipid synthesis and trafficking, including ACSL3, VPS13A and VPS13C. Furthermore, we show that ATG9A directly interacts with VPS13A and forms a complex that is distinct from the ATG9A–ATG2A complex.

## Teaser

**Highlighted Article:** Interactomics analysis of ATG9A uncovers lipid transport and homeostasis proteins as major hits, leading to the characterization of the ATG9A–VPS13A complex.

## INTRODUCTION

Macroautophagy, hereafter referred to as autophagy, is a catabolic process whereby protein aggregates or damaged organelles are cleared and recycled. Autophagy is classically initiated by nutrient deprivation. This causes the inhibition of mTOR, leading to the activation of the ULK complex and the class III phosphatidyl inositol 3 kinase (PIK3C3) complex I, ending downstream in membrane remodelling and the formation of a phagophore (Mercer et al., 2018; Morishita and Mizushima, 2019; Nishimura and Tooze, 2020).

Although the pathway is relatively well understood, the exact nature of autophagosome initiation and nucleation is still enigmatic (Melia et al., 2020). Recent studies have implicated ATG9A vesicles as the seed for the phagophore (Sawa-Makarska et al., 2020; Olivas et al., 2023), although an alternative or possibly complementary hypothesis is that ATG9A vesicles make transient contact with the phagophore to deliver the required proteins and lipids (Orsi et al., 2012; Judith et al., 2019; Karanasios et al., 2016; Koyama-Honda et al., 2013).

Autophagy relies on a network of specific ATG proteins, of which ATG9A and its paralogue ATG9B are the only transmembrane proteins. Recent research has shown that ATG9A functions as a lipid scramblase and falls into a unique class of transporters (Maeda et al., 2020; Matoba et al., 2020). ATG9A has four transmembrane domains and two helices that are perpendicular to the membrane (Maeda et al., 2020; Matoba et al., 2020; Guardia et al., 2020). The protein is divided roughly in half between its N-terminal core domain and its C-terminal domain, the latter of which is largely unstructured and intrinsically disordered.

Our own research, combined with others, has shown that ATG9A forms a complex with ATG2A (Gomez-Sanchez et al., 2018; van Vliet et al., 2022; Ghanbarpour et al., 2021) and that disrupting this complex inhibits autophagy (Gomez-Sanchez et al., 2018; van Vliet et al., 2022). ATG2A, and its paralogue ATG2B, are proteins that harbour a long, hydrophobic cavity, which is thought to function as a bulk lipid transport tunnel transporting lipids to the growing phagophore (Valverde et al., 2019; Osawa et al., 2019). This new class of lipid transporters, also termed the repeating β-groove (RBG) superfamily (Levine, 2022; Neuman et al., 2022), also includes the VPS13 family of proteins. The four isoforms of VPS13, VPS13A, VPS13B, VPS13C and VPS13D all share some similarity with ATG2 proteins (Kumar et al., 2018; Ugur et al., 2020).

Recent studies have started to shed a light on possible roles for ATG9A beyond autophagy, including in plasma membrane repair and lipid droplet biogenesis, among others (Mailler et al., 2021; Claude-Taupin et al., 2021). Our aim was to investigate whether possible new interactors of ATG9A could help explain these autophagy-independent functions.

## RESULTS AND DISCUSSION

**Fig. 1.**
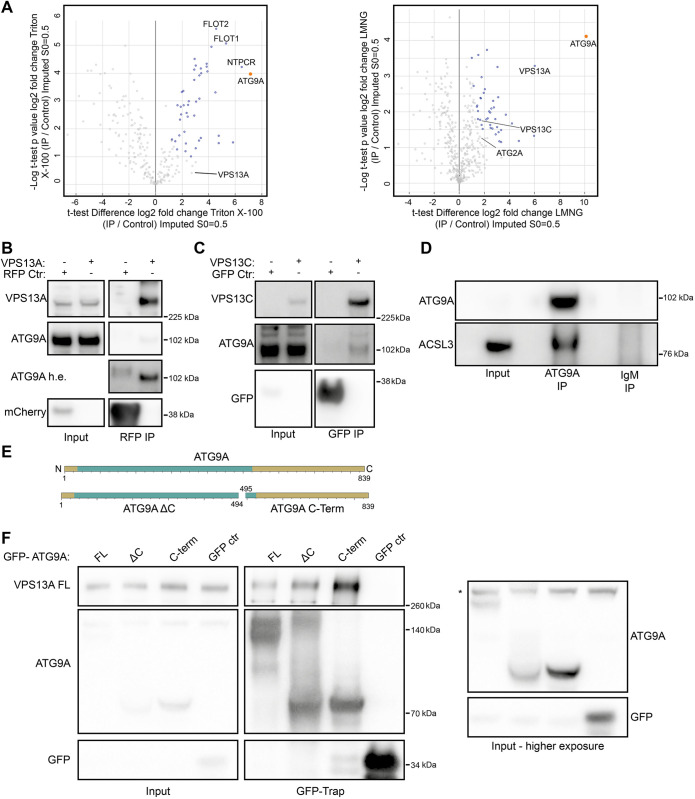
**Identification of new ATG9A-interacting proteins.** (A) Volcano plots of protein hits uncovered through mass spectrometry analysis by immunoprecipitating ATG9A after Triton X-100 or LMNG solubilization. The orange datapoint is ATG9A. Datapoints in blue are significantly enriched proteins as determined by an unpaired two-tailed *t*-test. Flotilin-1 (FLOT1), flotilin-2 (FLOT2), cancer-related nucleoside-triphosphatase (NTPCR), vacuolar protein sorting 13 homolog A and C (VPS13A and VPS13C) and ATG2A are indicated. (B) Immunoblot showing co-IP from cells of endogenous ATG9A using VPS13A^mCherry (tagged internally to preserve function). 1.25% of total lysate was loaded as input. The ATG9A h.e. blot shows the same ATG9A blot but with higher exposure. (C) Immunoblot showing co-IP from cells of endogenous ATG9A using VPS13C^Clover (tagged internally to preserve function). 1.25% of total lysate was loaded as input. (D) Immunoblot showing co-IP from cells of endogenous ACSL3 using anti-ATG9A antibodies. 1.25% of total lysate was loaded as input. (E) Scheme of ATG9A fragments used in F. Green depicts the N-terminal core domain, gold the rest of the protein sequence. (F) Immunoblot showing co-IP from cells expressing 3×Flag–VPS13A full length (FL) with GFP–ATG9A FL, ΔC or C-Term. 1.25% of total lysate was loaded as input. The asterisk (*) denotes a non-specific band. Results shown in this figure are representative of three repeats.

To get a more holistic view of the interaction network of ATG9A, we performed an immunoprecipitation (IP) of endogenous ATG9A, followed by mass spectrometry analysis. To preserve protein–protein interactions and achieve as much coverage as possible we used two different detergents, Triton X-100 and lauryl maltose neopentyl glycol (LMNG) (Fig. 1A; Fig. S1A). We utilized this approach because transient or stable overexpression of ATG9A can cause localization artefacts (van Vliet et al., 2023), leading to a possible distortion of the obtained interactome. In addition, we hypothesized that the use of Triton X-100 could destabilize the structure of ATG9A and thus compromise native interactions. In contrast, LMNG has been extensively used to successfully purify membrane proteins, including ATG9A, and has been shown to be efficient at protein extraction and stabilization (Guardia et al., 2020; Stetsenko and Guskov, 2017). As a control we immunoprecipitated with control IgM antibodies. Overall, our analysis detected a total of 236 proteins in the Triton X-100 condition and 431 in the LMNG condition. Of these hits, 46 proteins in the Triton X-100 and 42 proteins in the LMNG condition are statistically significantly enriched when pulling down for ATG9A (Fig. 1A; Table S1).

Among the hits uncovered by this method was ATG2A (although not significantly enriched), which has already been reported to interact with ATG9A (Ghanbarpour et al., 2021; van Vliet et al., 2022). Some of the top hits are indicated (Fig. 1A). Interestingly, we detected proteins involved in lipid homeostasis and transport, including VPS13A, VPS13C, ACSL1 and ACSL3 (Table S1). ACSL1 and ACSL3 are transmembrane proteins present in the endoplasmic reticulum (ER) or lipid droplet membrane (Poppelreuther et al., 2012). As such, they would not be expected to be abundant in the same membranes as ATG9A. This opens up the possibility that ATG9A could be part of a larger lipid synthesis and trafficking complex at the ER or lipid droplets, possibly including TMEM41b and VMP1 (Schutter et al., 2020; Ghanbarpour et al., 2021).

VPS13A was detected as a unique hit in both the Triton X-100 and LMNG conditions and was one of the top hits in the LMNG condition. ACSL1 and ACSL3 were only detected in the LMNG condition. We subsequently validated ACSL3, VPS13A and VPS13C using IP and co-IP experiments (Fig. 1B–D), indicating the robustness of the mass spectrometry dataset. The VPS13 family of proteins show similarity to ATG2 proteins and function as lipid transport proteins. Furthermore, yeast Vps13 has been increasingly linked with autophagy and phagophore expansion (Lei et al., 2022; Dabrowski et al., 2023), and thus we decided to explore the interaction between ATG9A and the VPS13 proteins in more detail. Our analysis detected both VPS13A and VPS13C, but only VPS13A was found in both Triton X-100 and LMNG datasets, and in both cases was more enriched than VPS13C. In addition, the IP between ATG9A and VPS13A was relatively more robust than VPS13C (Fig. 1D). We therefore decided to focus on VPS13A.

### VPS13A binds the C-terminus of ATG9A

Our previous work on the ATG9A–ATG2A complex showed that ATG2A bound the N-terminal core domain of ATG9A, reflected by an efficient IP and co-IP with the ATG9A ΔC-terminus (residues 1–494, ATG9A ΔC) (van Vliet et al., 2022). We hypothesized that VPS13A would have a similar binding mechanism and tested binding with the full-length ATG9A (residues 1–839, ATG9A FL), ATG9A ΔC-terminus (residues 1–494, ATG9A ΔC) and the ATG9A C-terminus (residues 495–839, ATG9A C-Term) (schematically depicted in Fig. 1E). Surprisingly, VPS13A was efficiently pulled down by both ATG9A ΔC-terminus and ATG9A C-Term (Fig. 1F).

### ATG9A binds the C-terminus of VPS13A

**Fig. 2.**
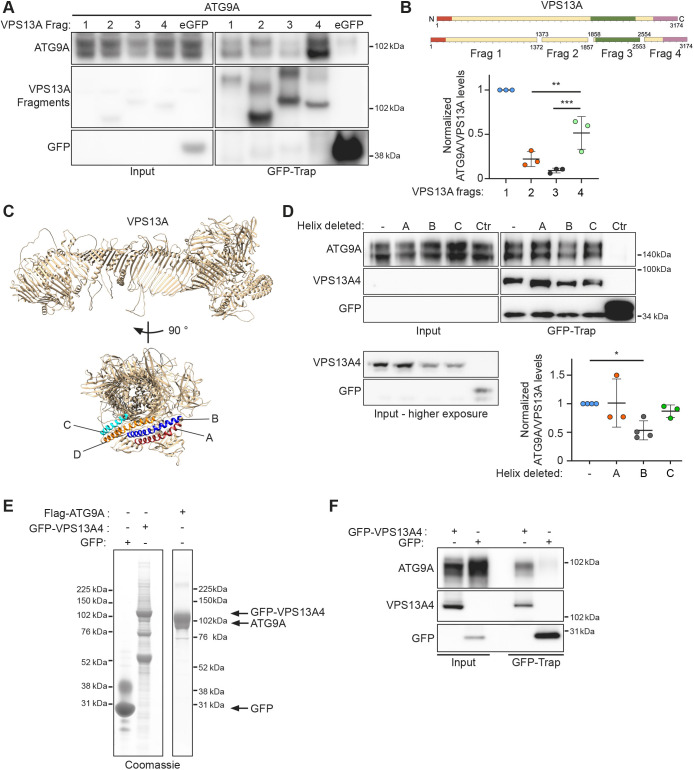
**Characterization of the ATG9A–VPS13A interaction.** (A) Immunoblot showing co-IP from cells expressing 3×Flag-ATG9A using the indicated expressed GFP–VPS13A fragments. 1.25% of total lysate was loaded as input. (B) Scheme of VPS13A fragments used in A. Orange depicts the chorein N domain, green the VAB domain and pink the PH and ATG­­­­_C domains. Also shown is the analysis of the experiment shown in A. Each datapoint in the graph represents the normalized ratio between ATG9A and VPS13A signals and depicts an independent experiment (*n*=3). The mean±s.d. is also indicated. **P<0.01, ***P<0.001 (one-way ANOVA with Dunnett's multiple comparison). (C) ColabFold predicted structure of human VPS13A in a ribbon representation. Identified α-helices are labelled A,B,C and D. (D) Immunoblot showing co-IP from cells expressing 3×Flag–ATG9A with GFP–VPS13A fragment 4 mutants (VPS13A4). 1.25% of total lysate was loaded as input. Each datapoint in the graph represents the normalized ratio between the ATG9A and VPS13A signals and depicts an independent experiment (*n*=3 for A and D, *n*=4 for B and Ctr). The mean±s.d. is also indicated. *P<0.05 (one-way ANOVA with Dunnett's multiple comparison). (E) Coomassie Blue stained SDS-PAGE gel of purified 3×Flag-VPS13A fragment 4 (VPS13A4) and 3×Flag–ATG9A. (F) Immunoblot of co-IP of purified VPS13A4 with purified ATG9A. Results shown in E and F are representative of three repeats.

Seeing that the ATG2 proteins are structurally related to the VPS13 family of proteins (Levine, 2022; Neuman et al., 2022), especially in the C-terminal regions, these observations led us to speculate that ATG9A could bind to VPS13 in a similar way. To test this, we made four fragments of VPS13A spanning residues 1–1372 (fragment 1, corresponding to a large part of the hydrophobic groove), 1373–1857 (fragment 2), 1858–2553 [fragment 3, roughly corresponding to the VPS13 adaptor-binding domain (VAB domain); Adlakha et al., 2022] and 2554–3174 [fragment 4, encompassing the pleckstrin homology (PH) domain and autophagy-related protein C terminal domain (ATG_C) domains] and tested which fragment(s) retained ATG9A binding (Fig. 2A,B). To our surprise, both fragments 1 and 4 retained substantial ATG9A binding (Fig. 2B). This is in contrast to our previous findings regarding ATG2A, where only the C-terminal fragment bound to ATG9A (van Vliet et al., 2022). To confirm the identified binding sites for ATG9A, we show that VPS13A fragment 4 retained the ability to bind to ATG9A FL, ΔC and the C-term (Fig. S1B). Using ColabFold (Mirdita et al., 2022), we were able to predict the structure of full-length VPS13A (Fig. 2C), in agreement with that from a previous publication (Guillen-Samander et al., 2022). We have previously shown that ATG9A binds to ATG2A through a C-terminal α-helix in the C-terminal localization region (CLR region, residues 1723–1829) of ATG2A (van Vliet et al., 2022). This region, in addition to the adjacent ATG_C region, shares substantial similarity to the C-terminal region of VPS13A. Using the predicted structure as a guide, we identified four α-helices in fragment 4, and in particular the C-terminal ATG_C region of VPS13A, that could be putative binding sites for ATG9A, labelled A to D (helix A, 2956-2987; helix B, 2991–3028; helix C, 2869–2893; helix D, 2895–2936) (Fig. 2C). After deleting each of these individually from the GFP-tagged fragment 4 and performing co-IP with ATG9A we observed an ∼50% reduction in ATG9A binding when helix B was deleted (Fig. 2D). Deletion of helix D caused an unexpected truncation of fragment 4, possibly due to instability, and this mutant was not taken into account in our analysis (data not shown).

Next, we purified both GFP–VPS13A fragment 4 and Flag–ATG9A (Fig. 2E) and performed an *in vitro* IP (Fig. 2F). This result confirmed that ATG9A can bind VPS13A4 directly and indicates that ATG9A and VPS13A can form a complex distinct from the ATG9A–ATG2A complex.

### Cellular significance of the VPS13A C-terminus

**Fig. 3.**
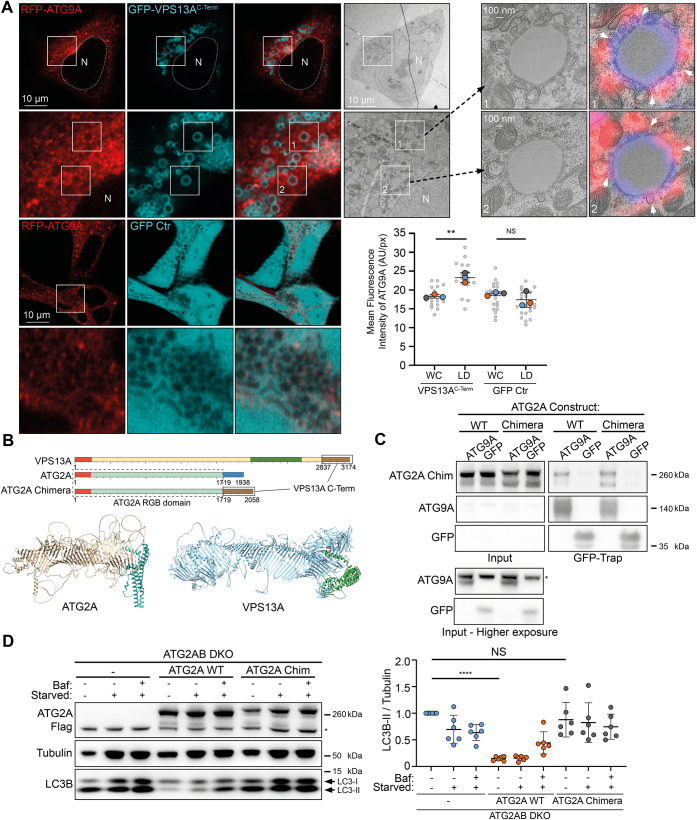
**Functional analysis of the ATG9A–VPS13A interaction.** (A) CLEM imaging of oleic acid-fed HEK293A cells expressing RFP–ATG9A (red) and GFP–VPS13A^C-Term^ (cyan). A low magnification TEM image of a fluorescently imaged cell is shown. Insets numbered 1 and 2 show high magnification images. White arrowheads depict locations where the limiting membrane of a lipid droplet makes close contact with RFP–ATG9A positive membranes. Each large datapoint in the graph depicts the average mean fluorescence intensity of RFP–ATG9A per region (WC, whole cell; LD, lipid droplets), and represents an independent experiment (*n*=3), with smaller grey datapoints representing all the technical replicates (AU, arbitrary units). The mean±s.d. is also indicated. **P<0.01; NS, not significant (one-way ANOVA with Dunnett's multiple comparisons test). (B) Scheme of VPS13A and ATG2A. Orange depicts the chorein N domains and forest green the VAB domain. Brown depicts the VPS13A C-terminal domain (encompassing PH and ATG_C domains). Blue depicts the ATG2A C-terminal domain (encompassing CLR and ATG_C domains). The RGB domain of ATG2A is teal. Chimera ATG2A is composed of the RGB domain of ATG2A (sequence 1–1719) and the C-terminus of VPS13A (sequence 2837–3174). Also depicted are the predicted structures of human ATG2A and VPS13A in a ribbon representation, with the C-terminal sequences that were exchanged depicted in cyan and green, respectively. (C) Immunoblot showing co-IP from cells stably expressing ATG2A WT and ATG2A–VPS13A chimera using GFP–ATG9A or GFP control. 1.25% of total lysate was loaded as input. The asterisk (*) denotes a non-specific band. Results shown in C are representative of three repeats. (D) Representative immunoblot of ATG2AB DKO cells stably expressing either empty vector (–), ATG2A WT or ATG2A–VPS13A chimera (ATG2A Chim). Cells were in either full medium, starvation medium (starved) or starvation medium with Bafilomycin A1 (Baf) for 3 h and analyzed for LC3B. Tubulin was used as a loading control. Each datapoint in the graph represents the normalized ratio between LC3B-II and tubulin signals and depicts an independent experiment (*n*=6). The mean±s.d. is also indicated. ****P<0.0001; NS, not significant (one-way ANOVA with Dunnett's multiple comparisons test). The asterisk (*) denotes a non-specific band.

When a smaller piece of VPS13A fragment 4 (VPS13A^C-Term^), containing the PH and ATG_C domains and the ATG9A-binding site, spanning residues 2752–3174, is expressed in cells fed with oleic acid to induce lipid droplets, it was enriched around lipid droplets (Fig. 3A), as shown before for full-length VPS13A (Kumar et al., 2018). We used this observation to explore whether VPS13A^C-term^ at lipid droplets was able to recruit RFP–ATG9A (Fig. 3A). When expressing GFP–VPS13A^C-term^ in cells stably expressing RFP–ATG9A, we saw a significant increase of RFP–ATG9A fluorescence at lipid droplets when compared to total cell fluorescence of RFP–ATG9A. This increase was absent when expressing only GFP (Fig. 3A). We performed correlative light and electron microscopy on cells expressing GFP–VPS13A^C-term^ and RFP–ATG9A and observed the presence of vesicles adjacent to lipid droplets (Fig. 3A).

Seeing that the C-termini of VPS13A and ATG2A have areas of similarity, in addition to both being able to bind ATG9A, we wondered whether they could broadly fulfil similar roles in relation to autophagy and ATG9A binding. To test this, we used alphafold and our colabfold predictions to identify where the repeating β-groove (RGB) domain for both proteins ended and replaced the entire C-terminal domain of ATG2A (residues 1721–1938, starting after the RGB domain ends and encompassing the CLR and ATG_C domains), with the C-terminal domain of VPS13A (residues 2837–3174, starting after the RGB domain ends) (schematically depicted in Fig. 3B). We first confirmed that the newly created ATG2A–VPS13A chimera retained ATG9A binding when compared to ATG2A wild-type (WT) protein (Fig. 3C). We next asked whether the ATG2A–VPS13A chimera was able to restore autophagic flux in cells with double knockout of ATG2A and ATG2B (ATG2AB DKO cells). Strikingly, although ATG2A WT, stably expressed in ATG2AB DKO cells, was able to rescue autophagic flux, the ATG2A–VPS13A chimera was not (Fig. 3D), indicating the presence of a unique property in the ATG2A C-terminus that is required for functional ATG9A–ATG2A complex formation and activity in autophagy. This result opens up multiple hypotheses. There is a possibility that adding the VPS13A C-terminus to ATG2A destabilizes the hydrophobic groove and thus prevents lipid trafficking. It is also possible that the C-terminus of VPS13A binds to ATG9A differently from that of ATG2A, thereby forming a non-functional ATG9A–ATG2A–VPS13A chimeric complex. A third, similar option could be that by replacing the ATG2A CLR, ATG9A can no longer make a stable connection with the hydrophobic groove of ATG2A, thus preventing lipid transfer into the ATG9A containing membrane. Follow-up studies will be required to further investigate the residues and mechanism of the ATG9A–VPS13A complex binding, and its role in autophagy and beyond.

Taken together, this study uncovers part of the interactome of ATG9A using IP of endogenous ATG9A and subsequent mass spectrometry analysis. Through this analysis, we discovered that various lipid metabolic proteins, including ACSL1, ACSL3, VPS13A and VPS13C, co-precipitated with ATG9A. The VPS13 proteins, like ATG2A, are part of the RBG family of proteins that contain a long hydrophobic groove and can mediate bulk lipid transfer between membranes. In this study, we identify VPS13A and VPS13C as interactors of ATG9A and have elucidated some details of the ATG9A–VPS13A complex. Unlike ATG2 proteins, VPS13A and VPS13C both have an N-terminal FFAT motif, which binds VAP proteins in the ER and thus anchors the N-terminus of VPS13A and VPS13C near the ER membrane. We identify here that ATG9A binds to the C-terminus of VPS13A and possibly the N-terminus, and not through its VAB domain. Using the colabfold structure predictions, and comparisons with our previous ATG2A-binding data and deletion mutants, we mapped the putative ATG9A-binding site in VPS13A to one of the C-terminal α helices. This putative ATG9A-binding site, which we termed helix B, is in the ATG_C region of VPS13A. A site in this helix was also found to be responsible for VPS13A recruitment to mitochondria (Guillen-Samander et al., 2022), leading us to hypothesize that helix B might mediate binding to various interaction partners/membranes.

### Limitations of the study

In our VPS13A fragment IP and ATG9A co-IP experiment (Fig. 2) expression levels of the different fragments was challenging to equalize. Especially fragment 1, which encompasses most of the hydrophobic groove, has very poor expression relative to the other fragments, and as such normalizing to expression levels in the case of fragment 1 might not be desirable.

Using ColabFold to predict the structure of VPS13A was successful; however, as with all predictions it is not a solved structure. The sequences we chose to delete corresponded to α-helices in the predicted structure but these might not reflect the true structure.

Our experiments using the ATG2A–VPS13A chimera failed to rescue autophagic flux to WT levels. However, even though the entirety of the hydrophobic groove which transports lipids was unchanged, replacing the ATG2A C-terminus with the VPS13A C-terminus could affect intrinsic lipid trafficking of the protein. This would then affect autophagic flux regardless of the ATG9A binding modality.

Purifying human full-length VPS13A proved challenging, and we were unable to get enough protein to perform an *in vitro* IP with purified ATG9A. Our interpretation that ATG9A and VPS13A interact directly is based on the *in vitro* IP of ATG9A and VPS13A fragment 4. However, it is possible that this interaction is different when using full-length VPS13A.

## MATERIALS AND METHODS

### Cell lines and transfection

HEK293A cells were provided by Cell Services of the Francis Crick Institute and tested for contamination regularly. Cells were cultured in a humidified incubator at 37°C in 10% CO_2_ in full medium [Dulbecco's modified Eagle's medium (Merck, cat. no. D6429) supplemented with 10% fetal calf serum (Thermo Fisher Scientific, cat. no. A5256701) and 4 mM L-glutamine (Merck, cat. no. G7513)]. Cells stably expressing RFP–ATG9A were generated previously (Orsi et al., 2012) and were maintained in the presence of G418 (Thermo Fisher Scientific, cat. no. 10131035) at 400 μg/ml. All plasmids were transfected using Lipofectamine 2000 (Thermo Fisher Scientific, cat. no. 11668019) according to the manufacturer's instructions. Autophagy was induced as previously described (van Vliet et al., 2022); cells were washed three times with Earle's balanced salt solution (EBSS; 1 g/l D-glucose, 6.8 g/l NaCl, 0.4 g/l KCl, 0.151 g/l CaCl_2_·2H_2_O, 0.2 g/l mM MgSO_4_·7H_2_O, 0.124 g/l NaH_2_HPO_4_·2H_2_O, and 2.2 g/l NaHCO_3_) and incubated in EBSS for 3 h. Where indicated, cells were treated with 100 nM Bafilomycin A1 (Thermo Fisher Scientific, cat. no. 328120001) for the specified time. HEK293A stable cells were maintained in full medium containing 1 μg/ml puromycin (Merck, cat. no. P8833). HEK293A ATG2A/B DKO cells were a kind gift from Prof. Thomas Melia, (Yale University, CT, USA). DNA plasmid amounts used were in the range of 1–6 μg per 10 cm dish plated to 80% cell confluency.

Stable cell lines were generated as described previously (van Vliet et al., 2022). To generate stable cell lines using the ATG2A/B DKO cell line, cell cultures were transduced with Lenti-virus containing empty PLVX-Puro (empty) vector (Takara Bio, cat. no. 632164), PLVX-Puro-ATG2A WT or PLVX-ATG2A-VPS13A chimera. The cell lines were selected with puromycin for 7 days, initially with 3 µg/ml, then 2.5 µg/ml and then maintained in 2.0 µg/ml. The lenti-viruses were generated by using the Lenti-X 293T cell line (Takara Bio, cat. no. 632180) transfected with the ATG2A PLVX-Puro constructs or empty vector (as above) using Lenti-X Packaging Single Shots (VSV-G) (Takara Bio, cat. no. 631275) consisting of Xfect transfection reagent premixed with VSV-G pseudotyped Lenti-X lentiviral packaging plasmids.

### Antibodies

The following primary antibodies were used for western blotting (WB) or IP: anti-Flag M2 (Sigma F3165, used at 1:1000 for WB), rabbit anti-ATG9A [Cancer Research UK (raised in-house), used at 1:1000 for WB], mouse anti-GFP [Cancer Research UK (raised in-house), clone 3E10, used at 1:1000 for WB], rabbit anti-mCherry (Evrogen, cat. no. AB233, used at 1:1000 for WB), rabbit anti-ACSL3 (Proteintech, cat. no. 20710-1-AP, used at 1:1000 for WB), rabbit anti-LC3B (Abcam, #ab51520, used at 1:1000 for WB), rat anti-tubulin (raised in-house, used at 1:2000 for WB), Armenian hamster anti-ATG9A (raised in-house, used for IP), control hamster IgM antibodies (Biolegend, cat. no. 401002, used for IP), HRP-conjugated secondary antibodies used for WB were from GE Healthcare [cat. no. NA931 (anti-mouse IgG), cat. no. NA934 (anti-rabbit IgG), used at 1:5000 for WB].

### Plasmids

VPS13A^mCherry and VPS13C^mClover3 were Addgene plasmids #118758 and #118760 (deposited by Pietro De Camilli; Kumar et al., 2018). Plasmids were generated through PCR using the in-fusion HD cloning kit (Takara Bio, cat. no. 639648), GFP-tagged plasmids were generated with the GFP C1 plasmid (Clontech) using either the EcoRI and XhoI or EcoRI and KpnI restriction sites. 3×Flag plasmids were cloned starting from the GFP C1 plasmid and, using the AgeI and BamHI restriction sites, 3×Flag with a linker sequence (GSGAGAGAGAILNSRV) along with the GFP C1 multiple cloning site were inserted. Full-length VPS13A was cloned sequentially using VPS13A^mCherry as a template, cloning both fragments of VPS13A flanking the mCherry sequence into the 3×Flag plasmid using restriction enzymes XhoI and SalI and SalI and BamHI. ATG2A Chimera was cloned sequentially, where the ATG2A C-terminus (residues 1721–1938) was first deleted from the previously made PLVX 3×Flag ATG2A plasmid using Q5 mutagenesis, leaving the glycine at position 1720 and flanked by an EcoRI site. EcoRI and BamHI were then used to insert the VPS13A C-terminus (2837-3174), leaving three amino acids to function as a linker between both regions. All plasmids can be obtained upon request.

### Immunoprecipitation and western blotting

Immunoprecipitation was undertaken as reported previously (van Vliet et al., 2022). Cells were lysed in ice-cold TNTE buffer (20 mM Tris-HCl pH 7.4, 150 mM NaCl, 1% w/v Triton X-100, 5 mM EDTA) modified TNTE buffer (20 mM Tris-HCl pH 7.4, 150 mM NaCl, 1% w/v Triton X-100, 5 mM EDTA, 0.5 mM TCEP, 5% glycerol) or LMNG buffer [20 mM Tris-HCl pH 7.4, 150 mM NaCl, 1% w/v lauryl maltose neopentyl glycol (LMNG), 5 mM EDTA] containing EDTA-free Complete Protease Inhibitor cocktail (Merck, cat. no. 5056489001). Lysates were cleared by centrifugation at 21,000 ***g*** and precleared with binding control agarose beads (Proteintech, cat. no. bab-20) for 1 h at 4°C. GFP-tagged proteins were immunoprecipitated using GFP-TRAP beads (Proteintech, cat. no. gta-20) and mCherry-tagged proteins with RFP-Trap (Proteintech, cat. no. rta-20) overnight at 4°C; 10 µl of bead slurry was used per 10 cm plate. Endogenous ATG9A was immunoprecipitated by coupling Armenian hamster anti-ATG9A or control hamster IgM antibodies to protein A Dynabeads^®^ (Thermo Fisher Scientific, cat. no. 10002D); 10 μg of antibody was coupled to 50 μl of bead slurry. Resin was washed four times with TNTE or LMNG buffer and bound protein was eluted with 2.5× Laemmli buffer at 65°C for 5 min before resolving by SDS-PAGE (4–12% Bis-Tris NuPAGE gels, Life Technologies) followed by transfer onto a PVDF membrane (Millipore, cat. no. IPVH00010).

After incubation with primary and secondary antibodies the blots were developed by Amersham ECL Western Blotting Detection Reagents (Cytiva, cat. no. RPN2106) or with Luminata Crescendo Western HRP substrate (Merck, cat. no. WBLUR0500). Densitometry was performed with ImageQuantTL software (GE Healthcare).

### Proteomics

Treated samples with two different detergents [1% Triton X-100 and 1% LMNG (Genron, cat. no. NG310)] were run 10 mm from the top on the SDS-PAGE gel. Gel bands were then excised and placed into separate protein LoBind Eppendorf tubes, de-stained with 50:50, 50 mM ammonium bicarbonate (AmBic) and acetonitrile (ACN). Gel bands were then dehydrated with 100% ACN followed by reduction with dithiothreitol (10 mM DTT) at 37°C for 30 min, then alkylated with iodoacetamide (55 mM IAM) for 20 min in the dark, and finally rehydrated with 50 mM AmBic, containing 100 ng trypsin (Pierce Trypsin Protease, MS Grade), and incubated at 37°C overnight. The recovered peptides were dried by vacuum centrifugation then re-solubilised in 0.1% formic acid prior to liquid chromatography (LC)-MS analysis.

Recovered peptides were transferred into a glass autosampler vial sample. Each sample was analysed in technical triplicate using a Thermo Fisher Scientific QExactive mass spectrometer coupled to an UltiMate 3000 HPLC system for on-line liquid chromatographic separation. The sample was initially loaded onto a C18 trap column (Thermo Fisher Scientific Acclaim PepMap 100; 5 mm length, 300 µm inner diameter) then transferred onto a C18 reversed phase column (Thermo Fisher Scientific Acclaim PepMap 100; 50 cm length, 75 µm inner diameter). Peptides were eluted with a linear gradient of 5–40% buffer B (80% ACN, 0.1% formic acid, 5% DMSO) at a flow rate of 250 nl min^−1^ over 35 min.

Higher energy collisional dissociation was selected as the activation method. Singly charged and unknown charge state precursor ions were not analysed. Full MS spectra were acquired in the orbitrap (*m*/*z* 300–1800; resolution 70k; AGC target value 1E6) with the MS/MS spectra of the ten most abundant precursors from the preceding MS survey scan then acquired (resolution 17.5k, AGC target value 1E5; normalized collision energy 28 eV; minimum AGC target 1E2). Selected precursors were dynamically excluded for 15 s.

Raw data files were processed on MaxQuant software (version 2.0.3.0). The LFQ algorithm and match between runs settings were selected. Enzyme specificity for trypsin was selected (cleavage at the C-terminal side of lysine and arginine amino acid residues unless proline is present on the carboxyl side of the cleavage site) and a maximum of two missed cleavages were allowed. Cysteine carbamidomethylation was set as a fixed modification, while oxidation of methionine and acetylation of protein N-termini were set as variable modifications. The peptide lists generated searched against the reviewed UniProt human proteome using the Andromeda search engine embedded in MaxQuant (Cox and Mann, 2008). MaxQuant also searched the same database with reversed sequences of 1% false discovery rate (FDR) at peptide and protein levels. A built-in database of common protein contaminants was also searched.

The ‘proteingroups.txt’ output file generated on MaxQuant was loaded in Perseus version 1.4.0.2. Contaminant and reverse protein hits were removed. LFQ intensities were log2 transformed.

For each sample, the triplicate was grouped. Data were filtered for at least two out of the three replicate LFQ intensity values in at least one group. Protein LFQ intensities were normalised, and missing values (NaN) were imputed from a normal distribution with default values (Tyanova et al., 2016). A protein was considered significantly differentially expressed when FDR<0.05.

### Protein expression and purification

Proteins were expressed and purified as reported previously (van Vliet et al., 2022). 3×Flag–eGFP–VPS13A and 3×Flag–His_6_–ATG9A were subcloned into the pcDNA3.1(+) vector (Genscript) for protein expression in Expi293 cells (Thermo Fisher Scientific). To transfect the cells, polyethyleneimine (linear MW 25000, Polysciences, cat. no. 23966-100) was mixed and incubated with plasmid DNA for 20 min in opti-MEM (Thermo Fisher Scientific) at a mass ratio of 3:1 (3×Flag–GFP–VPS13A) or 2:1 (ATG9A) and added to the cells in Expi293 expression medium (Thermo Fisher Scientific, cat. no. A1435101) at a cell density of 4×10^6^ cells/ml. The transfected cells were harvested after 72 h, washed in PBSA buffer, pelleted at 600 ***g***, frozen using liquid nitrogen and stored at −80°C until purification. Frozen cells expressing Flag–eGFP–VPS13A fragment 4 were thawed in VPS13 buffer (50 mM HEPES, pH 8, 500 mM NaCl, 10% w/v glycerol and 1 mM TCEP) supplemented with cOmplete EDTA free protease inhibitor tablets. The resuspended cells were lysed by an additional four cycles of freeze–thaw before centrifuging the lysate at 20,000 ***g*** for 20 min at 4°C. The supernatant was incubated with anti-DYKDDDDK G1 affinity resin (Genscript) for 4 h at 4°C with mixing before washing the resin with VPS13 buffer four times. Chaperone removal buffer (50 mM HEPES pH 8, 500 mM NaCl, 10% w/v glycerol, 1 mM TCEP, 2.5 mM ATP and 5 mM MgCl_2_), was added to the washed resin and incubated overnight at 4°C with mixing. The resin was washed again with VPS13 buffer and Flag–GFP–VPS13A4 was eluted by incubating the resin with 240 µg/ml Flag peptide dissolved in VPS13 buffer.

Cells expressing ATG9A were thawed and resuspended in 9A buffer (50 mM Tris-HCl pH 8, 200 mM NaCl) supplemented with 2× cOmplete EDTA free protease inhibitor tablets. A volume equal to this cell suspension of 2.4% w/v LMNG and cholesteryl hemisuccinate (CHS) mixed at mass ratio of 5:1 in 9A base buffer was added to the suspension to lyse the cells and solubilise ATG9A. Lysis was undertaken for 40 min at 4°C with agitation before centrifugation at 4000 ***g*** for 20 min to remove insoluble material. The supernatant was incubated with anti-DYKDDDDK G1 affinity resin for 1 h at 4°C with agitation before washing the resin with 9A wash buffer (50 mM Tris-HCl pH 8, 200 mM NaCl, 0.002% w/v LMNG:CHS) supplemented with 2× cOmplete EDTA free protease inhibitor tablets. Elution was done by incubating resin with 240 µg/ml Flag peptide in 9A buffer. Eluted ATG9A protein was then run on a HiLoad 16/600 Superose 6 pg (Sigma, cat. no. 29323952) size exclusion chromatography column.

For the immune precipitation experiments, 100 μg of purified 3×Flag–GFP–VPS13A was supplemented with detergent to a final LMNG:CHS (5:1) concentration of 0.002% and mixed with 10 μg of ATG9A at 4°C before adding GFP-Trap beads. Beads were washed four times with detergent buffer [VPS13 buffer supplemented with detergent to a final LMNG/CHS (5:1) concentration of 0.002%]. Proteins were eluted from the beads using 2.5× Laemmli buffer at 65°C for 5 min.

### Light microscopy imaging

Cells were grown and treated on Mattek # P35G-1.5-14-C dishes. Live-cell images were acquired using a Zeiss LSM 880 Airyscan Confocal microscope in Airyscan mode (×63 oil-immersion lens) and Zeiss ZEN imaging software. RFP–ATG9A mean fluorescence intensity was calculated using ImageJ. Briefly, either the region of lipid droplet clustering, or the whole cell was selected and mean fluorescence intensity of RFP–ATG9A was calculated in this area, averaged over multiple experiments and cells and plotted on a graph.

### Correlative light and electron microscopy

For correlative light and electron microscopy (CLEM), GFP–Flag–VPS13A^C-Term^ was transiently transfected in stably expressing mRFP–ATG9A HEK293A cells. Cells were grown on gridded dishes (Mattek # P35G-1.5-14-C-GRD) and incubated overnight with medium supplemented with 400 µM oleate (a kind gift from Max Gutierrez) to induce lipid droplet formation. Cells were then fixed in fixation buffer (8% formaldehyde, 2.5% glutaraldehyde and 0.1 M phosphate buffer, pH 7.4) added directly to the cells for 1 h. Fixation buffer was then removed and cells were washed twice with PBS allowing identification and imaging of cells of interest by phase contrast and fluorescence microscopy, using a 40× and 63× oil objective and taking 0.185-μm-thick *z*-stacks on a Zeiss LSM 880 microscope run in Airyscan mode. After imaging, cells were reincubated with fixation buffer overnight at 4°C. The samples were postfixed in 1% reduced osmium tetroxide, stained with tannic acid, dehydrated stepwise to 100% ethanol, and embedded in Epon using a Pelco Biowave. Serial sections (∼90 nm) were cut using a Leica UC7 ultramicrotome (Leica Microsystems, Milton Keynes, UK), collected on formvar-coated slot grids, and poststained for 10 min with 1% uranyl acetate and 5′ with Reynolds lead citrate. Grids were then imaged on a FEI Tecnai F20 electron microscope at 200 keV using a Falcon III detector. CLEM image overlays were produced using the ec-CLEM plugin in the Icy software (Paul-Gilloteaux et al., 2017).

### AlphaFold2-based predictions

Structures for two fragments of VPS13A (residues 1–2100 and residues 1021–3174) were generated using ColabFold v1.5.2 (https://colab.research.google.com/github/sokrypton/ColabFold/blob/main/AlphaFold2.ipynb) and predicted with an overall pLDDT (confidence value) of 72.5 and 74.5, respectively. The segments were stitched together using COOT (https://www2.mrc-lmb.cam.ac.uk/personal/pemsley/coot/) after deletion of the overlapping regions.

### Quantifications and statistical analysis

The statistical details of all experiments are reported in the figure legends and figures. Statistics were performed using GraphPad Prism 10 software (https://www.graphpad.com/), as detailed in figure legends. Given the assay characteristics and visual assessments, we made an assumption regarding data normality, which was not formally verified due to the limited sample size. The exact ‘*n*’ values are explicitly provided in the figure legends, and we denoted statistical significance using asterisks in the figures (*P<0.05, **P<0.01, ***P<0.001) when applicable. Sample sizes, ranging from three to six, were determined based on preliminary experiments and assay variability. These sample sizes are consistent with those reported in the literature using similar methodologies, and no data meeting acceptable experimental standards were omitted. Seeded cells were randomly assigned to experimental groups.

## Supplementary Material



10.1242/joces.261081_sup1 Supplementary information

Table S1. List of all identified proteins detected by Mass Spectrometry immunoprecipitated by ATG9A. Different tabs of the worksheet represent datasets obtained when cells were lysed using Triton X-100 or LMNG and a third tab combining both datasets (tab titled all proteins). Common contaminants were removed.
