# Supplementary material for: Exploring the ATG9A interactome uncovers interaction with VPS13A
Source: J Cell Sci. Author manuscript; Available in PMC 2024 Mar 6. (PMC10911177; doi:10.1242/jcs.261081)
Supplement: Supplementary Information [file EMS194369-supplement-Supplementary_Information.pdf]

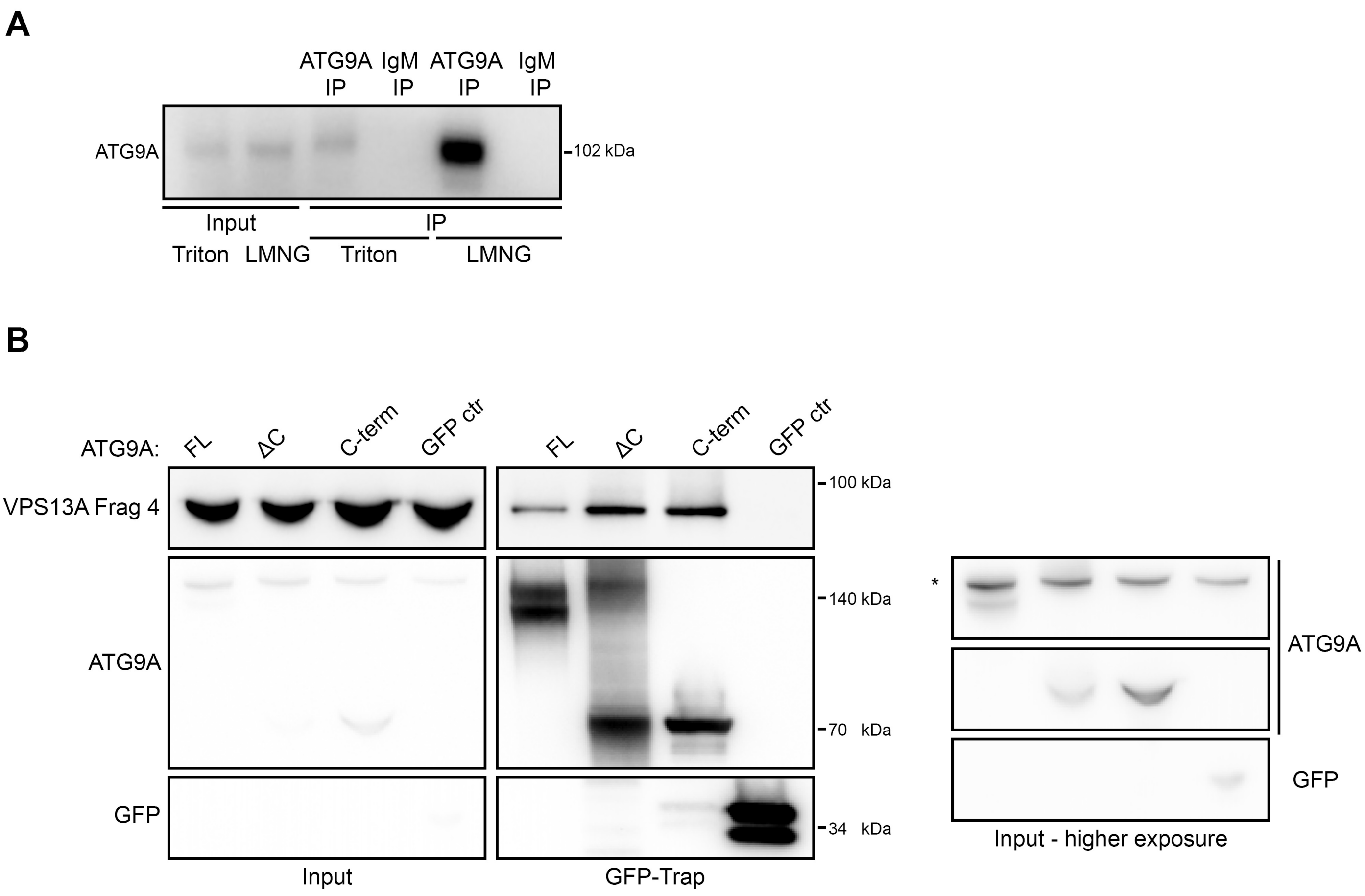

Fig. S1. (A) Western blot analysis of mass spectrometry samples immunoprecipitated using antibodies specific for ATG9A. (B) Immunoblot of co-IP performed from cell extracts of 3xFlag-VPS13A fragment 4 (VPS13A4) with expressed GFP-ATG9A FL, ΔC or C-Term. 1.25% of total lysate was loaded as input. *denotes aspecific band.

Journal of Cell Science • Supplementary information

Table S1. List of all identified proteins detected by Mass Spectrometry immunoprecipitated by ATG9A. Different tabs of the worksheet represent datasets obtained when cells were lysed using Triton X-100 or LMNG and a third tab combining both datasets (tab titled all proteins). For the Triton and LMNG tabs, the first eight columns (in blue) represent the Log2 LFQ intensity (Normalized intensity) of proteins found in each ATG9A and control condition (Each condition was analysed in triplicate), followed by their median value. The Score is the measure of the certainty of protein identification. The MS/MS counts are the peptide spectrum matches for the indicated protein. Razor+ unique peptides is total number of razor + unique peptides associated with the protein group (i.e. these peptides are shared with another protein group). The columns in yellow detail the statistical test values and whether the t-test performed for the log2 fold change between the bait and control condition is significant ($p<0.05$), when marked with '+', this particular protein was found significant. The remaining columns (in green) are protein IDs (Identifier(s) of protein(s) contained in the protein group), Majority protein IDs (These are the IDs of those proteins that have at least half of the peptides that the leading protein has) and protein/gene names (Name(s) of protein(s) or gene(s) associated to the protein(s) contained within the group). Common contaminants were removed.

Available for download at
https://journals.biologists.com/jcs/article-lookup/doi/10.1242/jcs.261081#supplementary-data
